# Cyp2aa9 regulates haematopoietic stem cell development in zebrafish

**DOI:** 10.1038/srep26608

**Published:** 2016-05-20

**Authors:** Jingying Chen, Jianbo He, Li Li, Deqin Yang, Lingfei Luo

**Affiliations:** 1 Key Laboratory of Freshwater Fish Reproduction and Development, Ministry of Education, Laboratory of Molecular Developmental Biology, School of Life Sciences, Southwest University, Beibei, 400715 Chongqing, China; 2 Department of Endodontics and Operative Dentistry, Chongqing Key Laboratory for Oral Diseases and Biomedical Sciences, The Affiliated Hospital of Stomatology, Chongqing Medical University, 401147 Chongqing, China

## Abstract

Definitive haematopoiesis occurs during the lifetime of an individual, which continuously replenishes all blood and immune cells. During embryonic development, haematopoietic stem cell (HSC) formation is tightly controlled by growth factors, signalling molecules and transcription factors. But little is known about roles of the cytochrome P450 (CYP) 2 family member in the haematopoiesis. Here we report characterization and functional studies of Cyp2aa9, a novel zebrafish Cyp2 family member. And demonstrate that the *cyp2aa9* is required for the HSC formation and homeostasis. Knockdown of *cyp2aa9* by antisense morpholino oligos resulted the definitive HSC development is defective and the Wnt/β-catenin activity becomes reduced. The impaired HSC formation caused by *cyp2aa9* morpholino can be rescued by administration of PGE2 through the cAMP/PKA pathway. Furthermore, the *in vivo* PGE2 level decreases in the *cyp2aa9* morphants, and none of the PGE2 precursors is able to rescue phenotypes in the Cyp2aa9-deficient embryos. Taken together, these data indicate that Cyp2aa9 is functional in the step of PGE2 synthesis from PGH2, thus promoting Wnt activation and definitive HSC development.

Haematopoietic stem cell (HSC), which are a self-renewing population of cells that continuously replenish all blood and immune cells during fetal and adult life^1^, first form in the definitive wave of haematopoiesis during vertebrate embryogenesis^2^. In mice, the original pool of HSCs is established in a complex developmental process that involves several anatomical sites^3^. The aorto-gonads-mesonephros (AGM) region where clusters of haematopoietic cells are found to associate with the ventral wall of dorsal aorta (VDA) has been widely believed as the initial site for HSC production^4^.

Zebrafish has been recognized as a powerful model organism for the study of haematopoiesis owing to its embryological and genetic advantages^5^. During the definitive wave, HSCs first emerge from the VDA at 28 hours post fertilization (hpf)^6^,^7^. These HSCs migrate to the caudal haematopoietic tissue (CHT) from two days post fertilization (dpf) on^8^,^9^. By 3 dpf, lymphopoiesis occurs in the thymus. One day later, the HSCs migrate to the kidney marrow, which is analogous to the bone marrow in mammals^10^,^11^,^12^.

The prostaglandins (PG), which is synthesized from arachidonic acid (AA), is an evolutionarily conserved regulator of HSCs^13^. Following the phospholipase-mediated release from phospholipids of the cell membrane, AA is sequentially converted to prostaglandin precursors G2 (PGG2) and H2 (PGH2) by cyclooxygenases Cox1/2^14^,^15^. These precursors are used to synthesize numerous prostanoids, including prostaglandin E2 (PGE2). In a recent chemical screen, PGE2 has been identified to regulate the HSCs homeostasis^16^. Further studies indicate that PGE2 can directly regulate the Wnt activity *in vivo* through the cAMP/PKA pathway^17^,^18^. The genetic interaction of PGE2 and the Wnt pathway has been demonstrated as a crucial signalling that regulates induction as well as homeostasis of HSCs^17^.

Cytochrome P450 (CYP) enzymes are involved in numerous detoxication and synthetic processes including generation of potent lipid mediators from endogenous substrates^19^. Biological functions of many CYPs become understood through identification of their substrates^21^. CYP isozymes can oxidize a spectrum of n-6 and n-3 polyunsaturated fatty acids (PUFA), such as retinoic acid, linoleic acid, eicosapentaenoic acid, docosahexenoic acid, and AA^20^. However, roles of CYP2s in the embryonic development remain largely unknown.

In this study, we have conducted a morpholino screen for all the 47 *cyp2* family genes in zebrafish to explore their functions in embryogenesis. In the *cyp2aa9* morphants, definitive HSC development is defective and the Wnt/β-catenin activity becomes reduced. The impaired HSC formation caused by *cyp2aa9* morpholino can be rescued by administration of PGE2 through the cAMP/PKA pathway. Furthermore, the PGE2 synthesis decreases in the *cyp2aa9* morphants, and none of the PGE2 precursors is able to rescue phenotypes in the Cyp2aa9-deficient embryos. So, we conclude that Cyp2aa9 functions at the step of PGH2 to PGE2 conversion, thus promoting Wnt activation and definitive HSC development.

## Results

### HSC development is defective in the *cyp2aa9* deficient embryos

**Figure 1:**
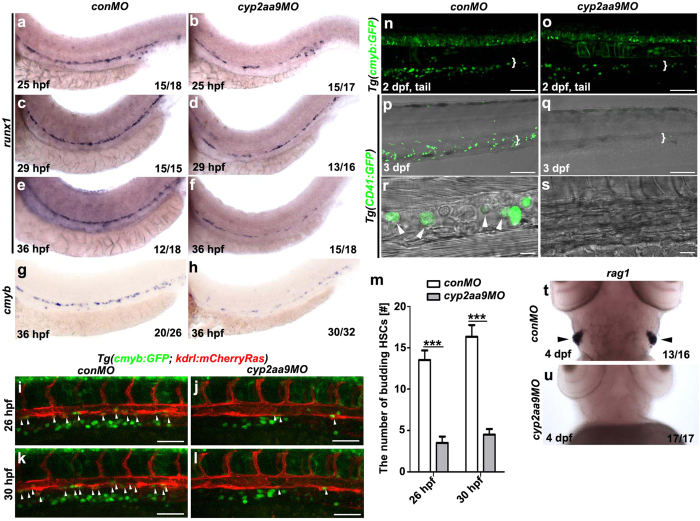
*cyp2aa9* is required for the HSCs formation. (**a**–**f**) In the *cyp2aa9* morphants, the expression of the definitive HSC precursors marker *runx1* became decreased in the ventral floor of the dorsal aorta from 25 hpf to 36 hpf. (**g**,**h**) The number of *cmyb*-expressing HSCs was significantly reduced in *cyp2aa9* morphants at 36 hpf. (**i**–**l**) In contrast to the control embryos at 26 hpf (**i**, n = 22/24) and 30 hpf (**k**, n = 23/24), the number of HSCs undergoing budding process from VDA (arrowheads) dramatically reduced in the *cyp2aa9* morphant (**j**, n = 21/25; **l**, n = 21/25; Scale bar, 50 μm). (**m**) Quantifications of the number of HSCs undergoing budding process (n = 8, mean ± SD, ***p < 0.001, Student’s t-test). (**n**,**o**) In CHT (white brackets), the number of *cmyb*:GFP-positive cells was significantly reduced in *cyp2aa9* morphants (27.6 ± 5.3, mean ± s.e.m., n = 6) comparing with the control (58.2 ± 4.1, mean ± s.e.m., n = 6). Scale bar, 100 μm. (**p**–**s**) Fluorecently labelled HSCs and DIC image showed the round-shaped HSCs (white arrowheads) were hardly detected in the CHT (white brackets) of *cyp2aa9* morphants under the *Tg*(*CD41:GFP*) background (**p**, n = 24/24; **q**, n = 25/29; scale bar, 100 μm). CHT regions were zoomed in and showed in the (**r**,**s**) (scale bar, 10 μm). (**t**,**u**) The expression of T-lymphocyte marker *rag1* in the thymus (arrowheads) dramatically decreased in the *cyp2aa9* morphants. (**a**–**s**) Lateral views, anterior left, dorsal up. (**t**,**u**) Ventral views, anterior up.

In a morpholino screen to explore roles of CYP2s in embryogenesis, we found that a morpholino oligo against the ATG-region of *cyp2aa9* (*cyp2aa9MO*) led to defects in definitive haematopoiesis. After injection of *cyp2aa9MO*, the precursors of definitive HSCs marked by *runx1* became decreased in the VDA from 25 hpf to 36 hpf (Fig. 1a–f). Expression of *cmyb*, which is enriched between the dorsal aorta and posterior cardinal vein as an indication of definitive HSC formation, was down-regulated in the *cyp2aa9* morphants at 36 hpf (Fig. 1g,h). Under the *Tg*(*cmyb: GFP, kdrl:mCherryRas*) transgenic background, real-time imaging showed that the number of *cmyb*+ HSCs budding from the VDA dramatically reduced in the *cyp2aa9* morphant from 26 hpf to 30 hpf (Fig. 1i–m, and see Supplementary Videos 1 to 2). By 2 dpf, the majority of the VDA-derived HSCs seed the CHT, an intermediate haematopoietic site analogous to the mouse fetal liver^2^. Under the *Tg*(*cmyb:GFP*) transgenic background, injection of *cyp2aa9MO* led to reduced HSCs in the CHT at 2 dpf (Fig. 1n,o, brace). At 3 dpf, the round-shaped HSCs were hardly detected in the CHT of the *cyp2aa9* morphants under the *Tg*(*CD41: GFP*) background (Fig. 1p–s). Generation of T-lymphocytes requires HSCs as precursors, providing a useful readout of HSCs^22^. Consistent with loss of HSCs in the CHT at the early stage (Fig. 1p–s), expression of the T-lymphocyte marker *rag1* in the thymus was nearly absent in the *cyp2aa9* morphants at 4 dpf (Fig. 1t,u). Taken together, these results demonstrated that loss of Cyp2aa9 impairs formation of HSCs during embryonic development.

To confirm the efficiency and specificity of the morpholino approach, *cyp2aa9MO* was co-injected with the *cyp2aa9-GFP* mRNA encoding the fusion protein. Expression of Cyp2aa9-GFP was specifically knocked down by the *cyp2aa9MO*, but not by a control morpholino (*conMO*) (See Supplementary Fig. S1a,b). To exclude the p53-mediated off-target effects of the morpholino which caused apoptosis^23^, a morpholino against *p53* (*p53MO*) was co-injected with *cyp2aa9MO* and no extra apoptosis induced by *cyp2aa9MO* was observed (See Supplementary Fig. S1c–e). Furthermore, although injection of the a morpholino-resistant *cyp2aa9* mRNA alone was ineffective to the body shape and HSC formation, it rescued the down-regulated *cmyb* expression in the *cyp2aa9* morphants (See Supplementary Fig. S2). These results confirm the efficiency and specificity of *cyp2aa9MO*, excluding the off-target or developmental delay effects. *In situ* hybridizations indicated diffused expression of *cyp2aa9* in the mesoderm and tail region at 20 hpf and 36 hpf (See Supplementary Fig. S3, arrowheads), spatially and temporally correlated with the budding of recognizable HSCs from VDA.

**Figure 2:**
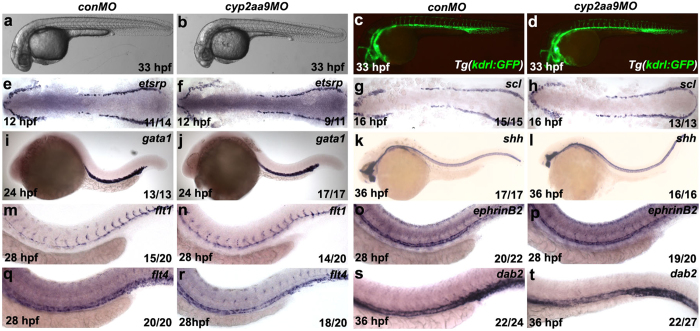
The non-HSC tissues are unaffected in the *cyp2aa9* morphants. (**a**–**d**) The *cyp2aa9* morphants in bright-field were morphologically normal (**a**, n = 110/110; **b**, n = 125/130) and with intact vasculature at 33 hpf under the *Tg*(*kdrl:GFP*) background (**c**, n = 21/21; **d**, n = 21/25). (**e**–**t**) The *cyp2aa9* morphants displayed normal expressions of the following tissue-specific markers: primitive haematopoiesis (*etsrp*, **e**,**f**; *scl*, **g**,**h**; *gata1*, **i**,**j**), notochord (*shh*, **k**,**l**), dorsal aorta (*flt1*, **m**,**n**; *ephrinB2*, **o**,**p**) and posterior cardinal vein (*flt4*, **q**,**r**; *dab2*, **s**,**t**). (**a**–**d**,**i**–**t)** Lateral views, anterior left, dorsal up. (**e**–**h**) Dorsal views, anterior left.

To further exclude that the impaired HSC development in the *cyp2aa9 *morphants is secondary to early developmental defects, we examined the integrity of haematopoietic and surrounding tissues according to morphology and expression of markers^24^. *cyp2aa9* morphants were morphologically normal at 33 hpf (Fig. 2a,b). And the *cyp2aa9* morphants displayed intact and functional vasculature as shown by beating hearts and circulating primitive non-HSC-derived erythroid cells under the *Tg*(*kdrl:GFP*) and *Tg*(*gata1:DsRed*) background (Fig. 2c,d; See Supplementary Fig S1f,g). Primitive haematopoiesis (*etsrp, scl, gata1*; Fig. 2e–j)^25^,^26^,^27^, notochord development (*shh*; Fig. 2k,l), dorsal aorta (*flt1, ephrinB2*; Fig. 2m–p)^28^,^29^, and the posterior cardinal vein (*flt4, dab2*; Fig. 2q–t)^28^ appear to be normal in the *cyp2aa9* morphants. Thus, defects in HSC formation caused by *cyp2aa9MO* were specific and not due to wholesale failures in the specification of primitive haematopoietic cells or the nearby tissues.

### *Cyp2aa9* regulates HSC formation and Wnt activity through the PGE2/cAMP/PKA pathway

**Figure 3:**
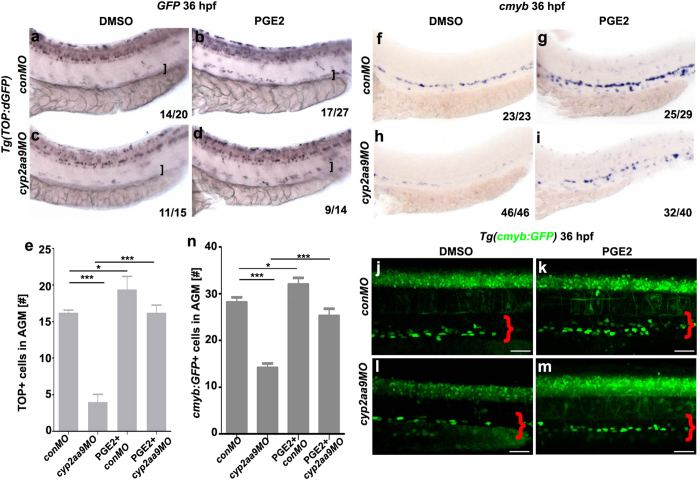
PGE2 rescues defective HSC formation and reduced Wnt/β-catenin activity caused by *cyp2aa9MO*. (**a**–**d**) Down-regulated Wnt/β-catenin activity in the VDA (brackets) caused by *cyp2aa9MO* was rescued by PGE2 as shown by WISH of GFP in the *Tg*(*TOP:dGFP*) Wnt-reporter line at 36 hpf. (**e**) Quantifications of total TOP-positive cells in the major trunk vessels (n = 6, mean ± SD). (**f**–**i**) Defective HSC formation in the *cyp2aa9* morphants was rescued by PGE2 as shown by *cmyb* expression. (**j**–**m**) Decrease in the number of HSCs in the *cyp2aa9* morphants was rescued by PGE2 as shown by the *cmyb:GFP* transgene. Scale bar, 50 μm. (**n**) Quantifications of the *cmyb:GFP*-positive cells in the AGM (n = 8, mean ± SD). *p < 0.05, ***p < 0.001, Student’s t-test. All the views of embryos are lateral, anterior left, dorsal top.

**Figure 4:**
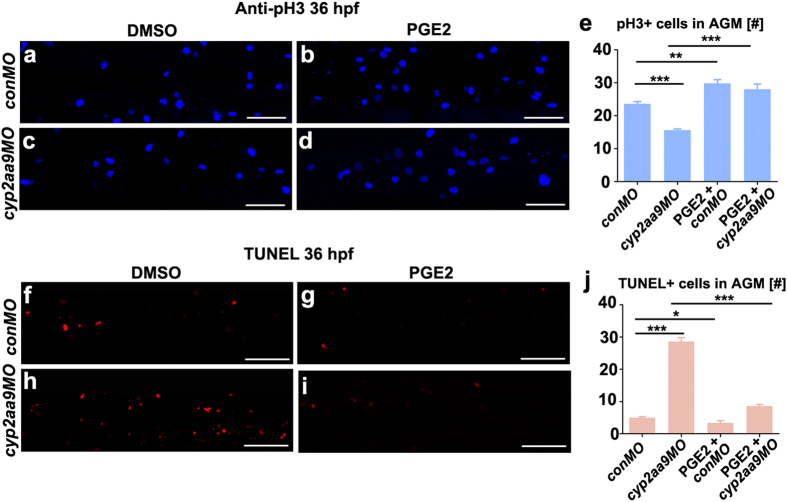
Defective proliferation and apoptosis caused by *cyp2aa9MO* were rescued by PGE2. (**a**–**d**) *cyp2aa9MO* led to reduced number of mitotic active cells at 36 hpf in the AGM as shown by pH3 antibody staining, which could be rescued by PGE2. (**e**) Quantification of pH3-positive cells in the AGM (n = 6, mean ± SD). (**f**–**i**) *cyp2aa9MO* led to increases in the number of apoptotic cells at 36 hpf in the AGM as shown by TUNEL assay, which could be rescued by PGE2. (**j**) Quantification of the number of apoptotic cells in the AGM (n = 6, mean ± SD). *p < 0.05, **p < 0.01, ***p < 0.001, Student’s t-test. All the views are exactly the AGM area shown in lateral, anterior left, dorsal top. Scale bar, 50 μm.

Canonical Wnt/β-catenin signalling and PGE2 are involved in the HSC specification and maintenance in vertebrates^17^. To examine whether Cyp2aa9 regulates the Wnt activity *in vivo*, we applied the *Tg*(*TOP:dGFP*) β-catenin responsive reporter line^30^. Decreases in the GFP expression in the VDA region of the *cyp2aa9* morphants were observed at 36 hpf, which could be rescued by the administration of PGE2 (Fig. 3a–e, brackets). PGE2 could also rescue the down-regulated *cmyb* expression and decreases in the number of HSCs caused by *cyp2aa9MO* (Fig. 3f–n). *Cyp2aa9MO* led to reduced number of mitotic active cells and increased number of apoptotic cells in the AGM region at 36 hpf, both of which could be efficiently rescued by PGE2 (Fig. 4a–j). These data indicate that defects in HSC development and Wnt activation in the *cyp2aa9* morphants can be rescued by PGE2.

**Figure 5:**
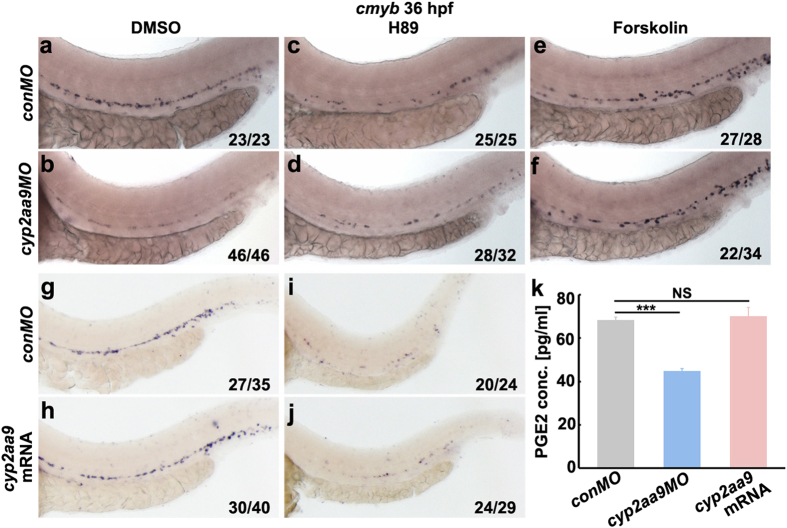
*Cyp2aa9* regulate HSC development through the PGE2/cAMP/PKA signalling. (**a**–**f**) Inhibition of PKA by H89 resulted in reduced *cmyb* expression in control, but did not exacerbate the HSC phenotype in *cyp2aa9* morphants. Enhancement of cAMP by Forskolin increased the *cmyb* expression in control, and rescued the defective HSC development in *cyp2aa9* morphants. (**g**–**j**) The inhibitory effects of H89 on HSC formation could not be rescued by the injection of *cyp2aa9* mRNA. (**k**) *In vivo* PGE2 concentration measured by ELISA assay. n = 3 tubes of lysates, mean ± SD, ***p < 0.001, NS, not significant, Student’s t-test. All the views of embryos are lateral, anterior left, dorsal top.

We then investigated whether Cyp2aa9 carried out its HSC regulatory function in parallel to PGE2 signalling or via PGE2. PGE2 has been demonstrated to regulate HSC formation and Wnt activity through cAMP and its downstream effector kinase PKA^17^. Reduced expression of *cmyb* in the VDA of *cyp2aa9* morphants was rescued by the treatment of Forskolin, a cAMP activator (Fig. 5a,b,e,f). Treatment of H89, a PKA inhibitor, resulted in defective HSC development, which could not be rescued by the injection of *cyp2aa9* mRNA (Fig. 5g–j), but did not exacerbate the HSC phenotype in the *cyp2aa9* morphants (Fig. 5c,d). To analyse whether Cyp2aa9 participates in the synthesis of PGE2 *in vivo*, we examined the PGE2 concentration using ELISA. Although *cyp2aa9* mRNA had no impact on the PGE2 synthesis, *cyp2aa9MO* led to decreases in the endogenous PGE2 concentration at 36 hpf (Fig. 5k), which became the direct evidence that Cyp2aa9 regulates PGE2 synthesis and acts upstream of PGE2 *in vivo*. These results demonstrate that Cyp2aa9 is required for PGE2 synthesis and regulates definitive HSC development through the PGE2/cAMP/PKA/Wnt cascade.

### Cyp2aa9 functions at the step of PGH2-to-PGE2 conversion

**Figure 6:**
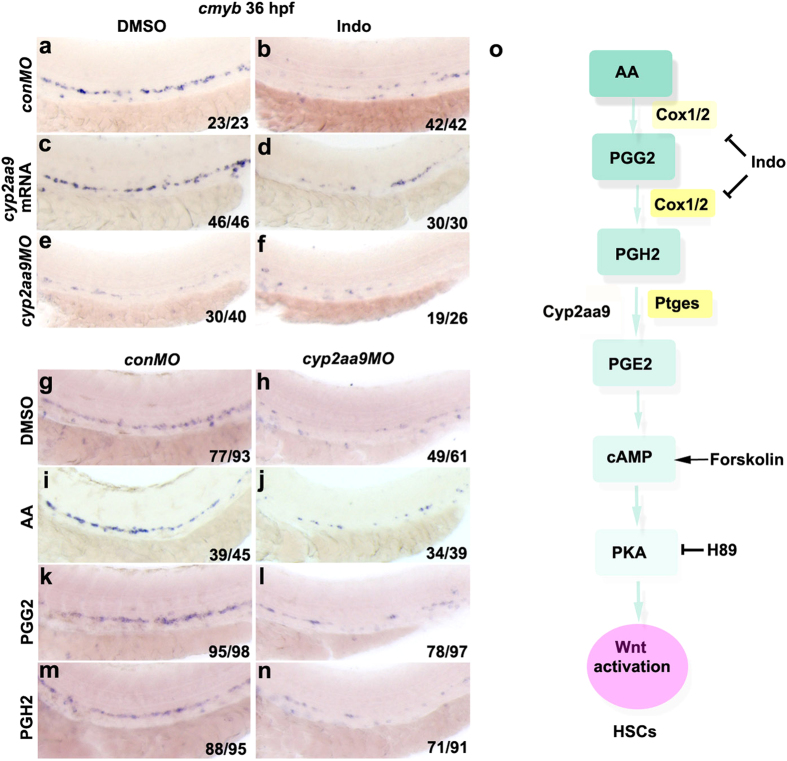
Roles of Cyp2aa9 in HSC development act at the step of PGH2-to-PGE2 conversion. (**a**–**f**) Down-regulation of *cmyb* expression caused by Indomethacin (Indo) could not be rescued by *cyp2aa9* mRNA, and could not be exacerbated by *cyp2aa9MO*. (**g**–**n**) The PGE2 precursors AA, PGG2 or PGH2 could not rescue the defective HSC formation in *cyp2aa9* morphants. (**o**) Illustration of the prostaglandin synthesis and PGE2/cAMP/PKA pathway involved in HSC development in zebrafish. Note that Cyp2aa9 acts at the step of PGH2-to-PGE2 conversion. Indo, non-selective cyclooxygenases inhibitor. Forskolin, cAMP activator. H89, PKA inhibitor. All the views of embryos are lateral, anterior left, dorsal top.

Steps of PGE2 synthesis include conversion of AA to PGG2 and in turn to PGH2 by Cox-1/2. Then, PGE2 is synthesized from PGH2 by prostaglandin E synthase (Ptges) (Fig. 6o). We investigated at which step Cyp2aa9 contributed to the prostaglandin metabolism. Impaired HSC development caused by the incubation with indomethacin (Indo), a non-selective cyclooxygenases inhibitor, could not be rescued by the injection of *cyp2aa9* mRNA (Fig. 6a–d), but did not exacerbate the HSC phenotype in the *cyp2aa9* morphants (Fig. 6e,f). Defective HSC development in the *cyp2aa9* morphants could be rescued by PGE2 (Fig. 3f–i), but not by any of its precursors AA, PGG2, or PGH2 (Fig. 6g–n), indicating that Cyp2aa9 is non-functional without production of PGH2. All these results suggest that Cyp2aa9 carries out its HSC regulatory functions at the step PGH2-to-PGE2 conversion (Fig. 6o).

## Discussion

This study reveals roles of a cytochrome P450 (CYP) member Cyp2aa9 in HSC development through promotion of PGE synthesis in zebrafish. Previous studies have shown that activation of Wnt signalling is required for HSC development^31^,^32^,^33^, and PGE2 interact with the Wnt pathways to regulate survival and proliferation of HSCs^17^. Our work found reduced Wnt activity and PGE2 synthesis in the *cyp2aa9* morphants, suggesting a model that Cyp2aa9 acts upstream of PGE2 signalling to mediate the full effects of Wnt activation in HSC development.

The regulatory role of Cyp2aa9 in PGE2 synthesis and HSC development requires the prostaglandin precursor PGH2. However, mechanisms underlying regulation of PGE2 synthesis by Cyp2aa9 remain unclear. Currently, there is no metabolic study characterizing the activity of Cyp2aa9 with potential substrates and metabolites^34^,^35^. The predicted Cyp2aa9 protein structure contains two transmembrane domains. According to the crystal structure of Cyp2aa1, which has been constructed based on crystal structures of its closely related mammalian CYP2s family, the most variable region within the Cyp2aa cluster occurs close to the substrate access channels^35^. These structural information suggests that different Cyp2aa members indeed obtain differences in substrate specificity, and these differences are possibly controlled by the variable residues near the entrances of substrate channels. Although we cannot conclude that PGH2 is the endogenous, direct substrate of Cyp2aa9, this could be a possible mechanism through which Cyp2aa9 regulates PGE2 synthesis. Another enzyme Ptges has been demonstrated to convert PGH2 to PGE2, and the relationship between Cyp2aa9 and Ptges remains unclear. The second possible mechanism is that Cyp2aa9 plays roles in the prostaglandin metabolism in an indirect manner, for example acting as the co-enzyme of Ptges. These hypotheses and detailed working mechanisms of Cyp2aa9 need further investigations.

In zebrafish, the Cyp2aa family consists of 10 genes that are tandemly duplicated in a locus on chromosome 23. However, this genomic locus share poor synteny with mammalian CYP2 genes^34^,^35^, and the different Cyp2aa members may have distinct substrate specificities^32^. Previous studies have demonstrated that the Cyp2aas transform a broad range of xenobiotic substrates^35^,^36^, whereas little is known regarding their physiological functions. Our study has identified physiological roles of Cyp2aa9 in HSCs formation during embryonic development.

Cox1/2 are involved in the conversion of AA to PGH2, which is a common substrate for other prostanoids/prostaglandins (PGD2, PGE2, PGI2 and TXA2)^37^. Alterations in the Cox1/2 activity have an impact on endothelial-derived HSCs^16^. Similar to the expression pattern of *cox2* (ref. 16), *cyp2aa9* is also expressed in the mesoderm and tail region at 20 hpf and 36 hpf (See Supplementary Fig. S3, arrowheads). This expression pattern is spatially and temporally correlated with the budding of recognizable HSCs from VDA. The *cyp2aa9* morphants exhibit reduced numbers of the *runx1*-positive cells and budding HSCs, in accordance with the regulatory roles of Cox2 in HSC development. Although HSC proliferation and survival are defective in the *cyp2aa9* morphant (Fig. 4a–j), the budding process of residual HSCs still occur (Fig. 1i–m, and see Supplementary Videos 1 to 2). Our study has demonstrated the regulatory roles of Cyp2aa9 in HSC formation through regulation of PGE2 synthesis, providing further insights into the physiological functions of Cyp2aa family members and HSC development.

## Materials and Methods

### Ethics statement

All experimental protocols were approved by the School of Life Sciences, Southwest University (Chongqing, China), and the methods were carried out in accordance with the approved guidelines. The zebrafish facility and study were approved by the Institutional Review Board of Southwest University (Chongqing, China). Zebrafish were maintained in accordance with the Guidelines of Experimental Animal Welfare from Ministry of Science and Technology of People’s Republic of China (2006) and the Institutional Animal Care and Use Committee protocols from Southwest University (2007).

### Fish lines

The zebrafish transgenic lines *Tg*(*cmyb:GFP*)^11^, *Tg*(*TOP:dGFP*)^24^, *Tg*(*kdrl:GFP*)^38^, *Tg*(*gata1:DsRed*)^39^ and *Tg*(*CD41:GFP*)^40^, *Tg*(*kdrl:mCherryRas*)^41^ were previously described. Embryos were treated with 0.003% 1-phenyl-2-thiourea (PTU, Sigma) from 24 hpf to inhibit pigmentation.

### Morpholino and mRNA injections

The ATG-morpholino *cyp2aa9MO* (5′-CTTCAGGAGAGCCGTAAACATGATG-3′) and *conMO* (5′-CTaCAGGtGAGCgGTAtACAaGcTG-3′, lowercase letters denote mismatched bases) were synthesized (Gene-Tools, LLC). To generate the *cyp2aa9MO*-resistant mRNA, five nucleotides within the MO target site were changed without altering the encoding amino acids. The morpholino-resistant *cyp2aa9* mRNA were synthesized from the SacII-linearized *pCS2*(+) constructs using the Message mMachine kit (Ambion) as previously described^42^. 5 ng of *cyp2aa9MO* and *conMO*, and 70 pg of *cyp2aa9* mRNA were injected as previously described^42^.

### Whole mount *in situ* hybridizations

The plasmids for probe synthesis were amplified and sub-cloned into the pGEMT-easy vector (Promega). Digoxigenin-labelled probes were generated and *in situ* hybridization was performed as previously described^24^,^42^,^43^. Images were captured using a SteREO Discovery 20 microscope equipped with Axio Vision Rel 4.8.2 software (Carl Zeiss).

### TUNEL assay and antibody staining

TUNEL assay was carried out using the *In Situ* Cell Death Detection TMR red (Roche) according to the manufacturer’s instructions. The whole-mount antibody staining was performed as previously described^44^. For the permeability of the whole mount embryos, embryos were treated with Proteinase K (10 μg/ml) at room temperature (RT) for 15 minutes and then with acetone at −20 °C for 30 minutes. Embryos were incubated with antibodies against phospho-histone 3 (pH3, 1:500; Millipore), then incubated with Alexa fluorescent-conjugated secondary antibodies (1:500; Invitrogen) diluted in the blocking solution at 4 °C overnight. After washed with the PBST, embryos were preceded for mounting and imaging. Images were captured by ZEN2010 software equipped on an LSM780 confocal microscope (Carl Zeiss).

### Treatment of embryos with chemicals

All analyses were performed at 36 hpf. Embryos were exposed to the following compounds in fish facility water from 10 hpf to 36 hpf^17^,^45^: 10 μM PGE2 (Sigma), 10 μM Indomethacin (Sigma); 0.5 μM Forskolin (Abcam, ab120058), 0.5 μM H89 (Abcam, ab120341), 20 μM Arachidonic acid (Sigma, A9673). DMSO carrier content was at 0.1%. Equivalent amount of DMSO was added to the media as control. For PGG2 (0.1 mg/ml, Cayman, 17010) and PGH2 (0.1 mg/ml, Santa Cruz, sc201266), embryos were injected with these chemicals in DMSO at the bud stage^46^.

### Fluorescent microscopy and *in vivo* time-lapse imaging

Antibody stained or live embryos were mounted in 1.2% low melting point agarose and imaged using ZEN2010 software equipped on an LSM780 confocal microscope (Carl Zeiss). For time-lapse live imaging, zebrafish embryos were raised in the presence of 0.003% PTU to avoid pigmentation. Time-lapse images were captured using a 20× water immersion objective mounted on the LSM780 confocal microscope equipped with heating stage to maintain 28.5 °C. Z image stacks were collected every 10 minutes, and three-dimensional data sets were compiled using ZEN2010 software (Carl Zeiss).

### ELISA measurement of PGE2

The embryonic lysates (50 embryos at 36 hpf in a tube) were prepared using the PGE2 ELISA kit (Invitrogen, KHL1701) according to the manufacturer’s instruction. The total PGE2 concentration was measured at a wavelength of 412 nm in the SPECTRA MAX 190 (Molecular Devices).

## Additional Information

**How to cite this article**: Chen, J. *et al*. Cyp2aa9 regulates haematopoietic stem cell development in zebrafish. *Sci. Rep.* **6**, 26608; doi: 10.1038/srep26608 (2016).

## Supplementary Material

Supplementary Information

Supplementary Information

Supplementary Information
