# Supplementary material for: Cyp2aa9 regulates haematopoietic stem cell development in zebrafish
Source: Sci Rep. 2016 May 20;6:26608. doi: 10.1038/srep26608 (PMC4873737; doi:10.1038/srep26608)
Supplement: Supplementary Information [file srep26608-s1.pdf]

Supplementary Information

Cyp2aa9 regulates haematopoietic stem cell development in zebrafish

Jingying Chen[1], Jianbo He[1], Li Li[1], Deqin Yang[2] & Lingfei Luo[1]

[1]Key Laboratory of Freshwater Fish Reproduction and Development, Ministry of Education, Laboratory of Molecular Developmental Biology, School of Life Sciences, Southwest University, Beibei, 400715 Chongqing, China

[2]Chongqing Key Laboratory for Oral Diseases and Biomedical Sciences, The Affiliated Hospital of Stomatology, Chongqing Medical University, 401147 Chongqing, China

Correspondence and request for materials should be addressed to L.L. or D.Y. (e-mail: lluo@swu.edu.cn, yangdeqin@gmail.com)

Supplementary Data:

Supplementary Figure S1 to S3

Supplementary Video Legends

Supplementary Materials and Methods

Supplementary Videos 1 to 2

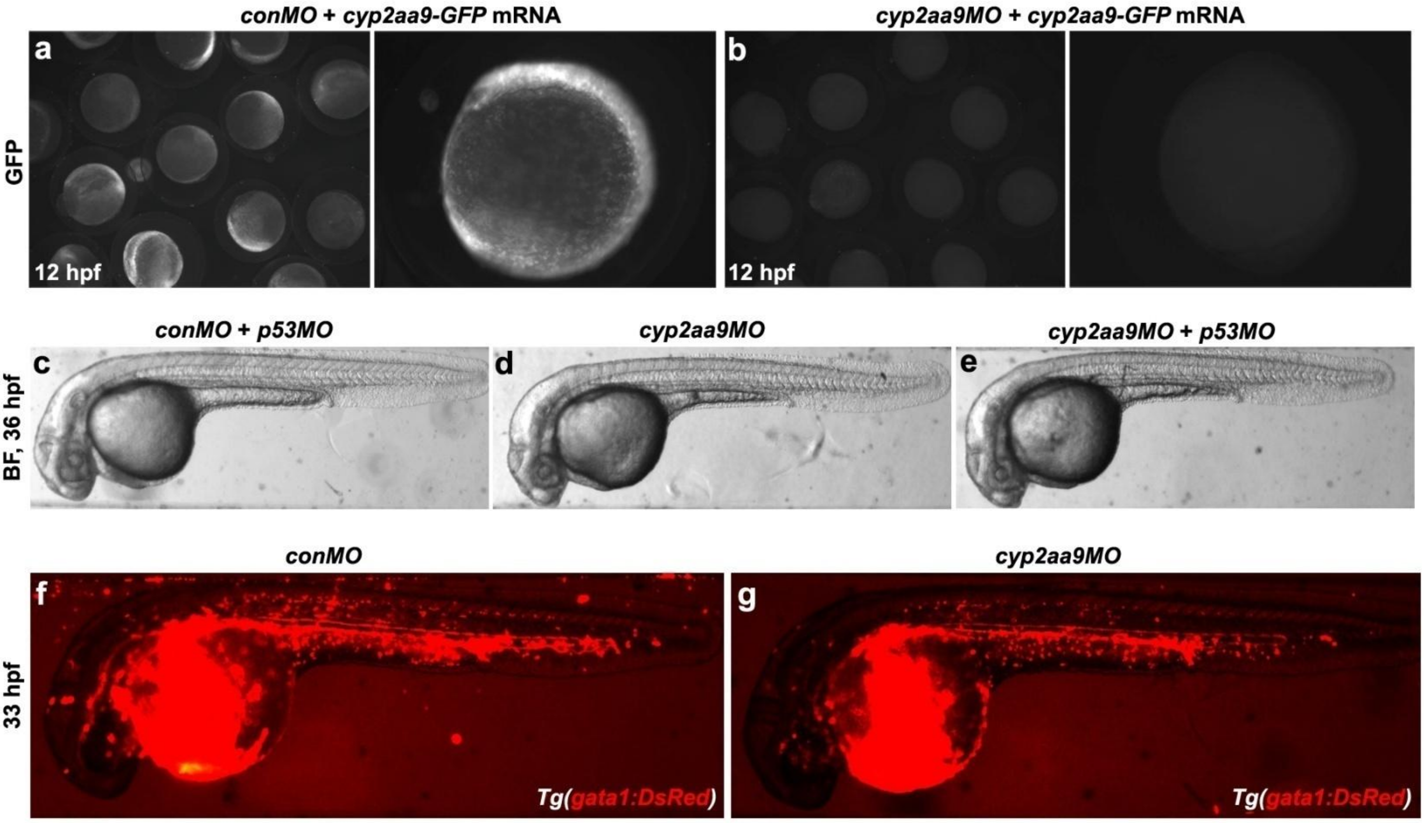

Supplementary Figure S1 (related to Figure 1). Effects of c*yp2aa9MO* is specific.

(**a**-**b**) Translation of *cyp2aa9-GFP* mRNA was inhibited by *cyp2aa9MO*, but not *conMO*. (**c**-**e**) *cyp2aa9MO* did not lead to obvious cell apoptosis as indicated by the co-injection of *p53MO*, excluding off-target effects of *cyp2aa9MO* (**c**, n=30/31; **d**, n=24/25; **e**, n=26/27). (**f**-**g**) The *cyp2aa9* morphants exhibited normal blood circulation under the *Tg(gata1:DsRed)* background (**f**, n=24/24; **g**, n=23/25). **c**-**f**, lateral views, anterior left, dorsal top.

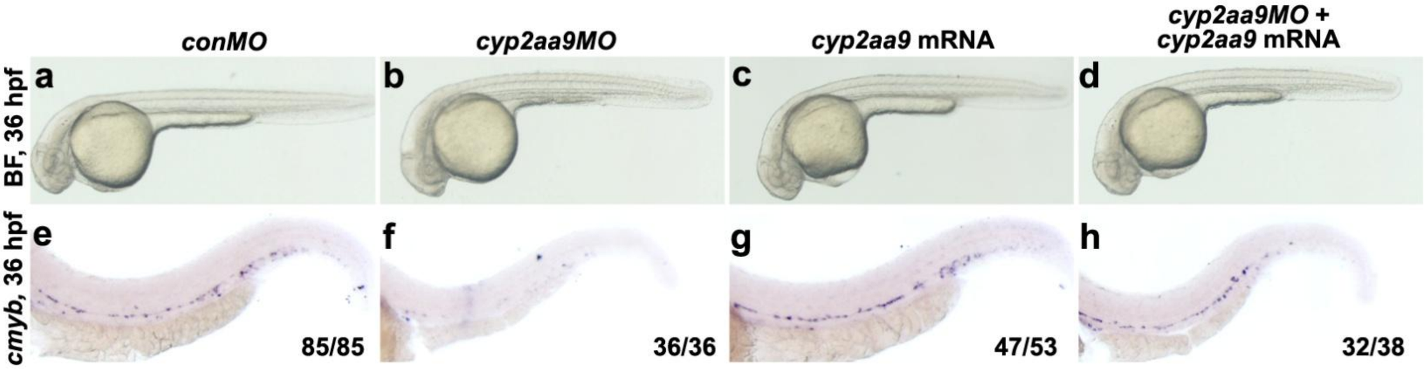

Supplementary Figure S2 (related to Figure 1). Defective *cmyb* expression in the *cyp2aa9* morphants was rescued by the *cyp2aa9* mRNA. (**a-h**) As indicated by the *cmyb* expression, defective HSC development in the *cyp2aa9* morphants was rescued by the *cyp2aa9* mRNA. lateral views, anterior left, dorsal top.

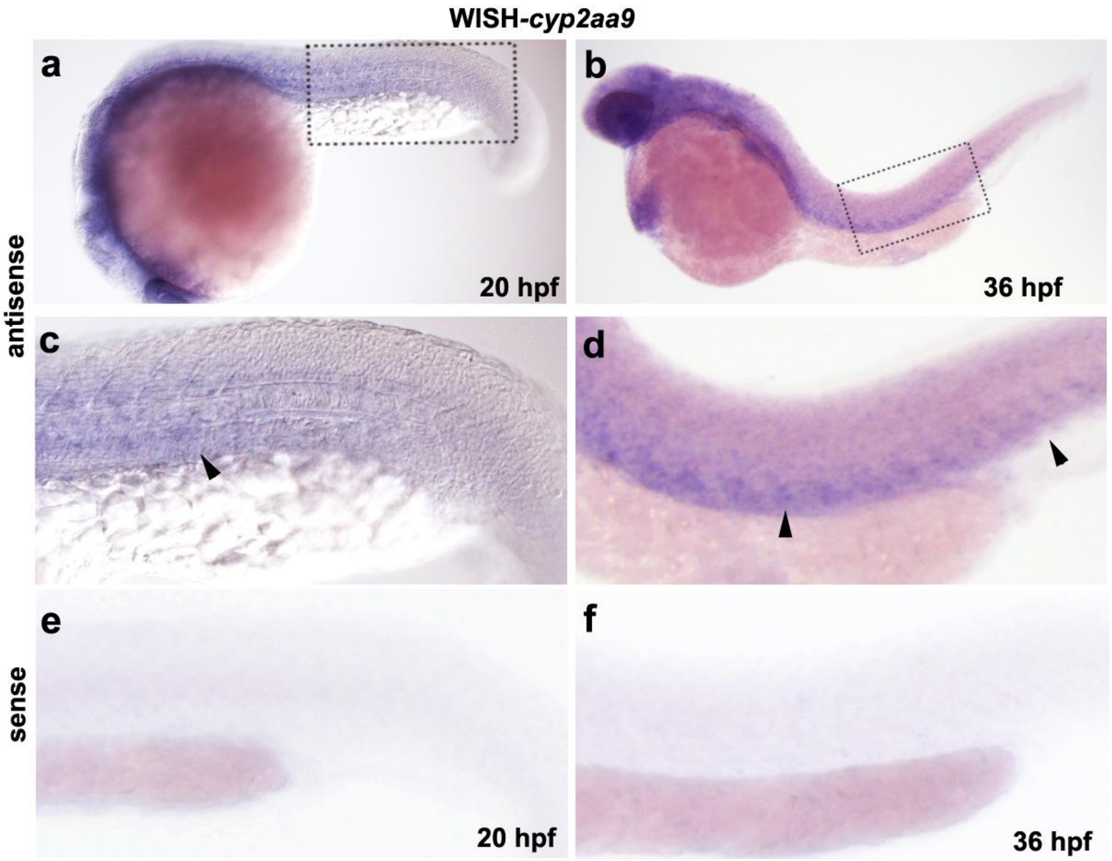

Supplementary Figure S3 (related to Figure 1). Expression pattern of *cyp2aa9* in zebrafish embryos. (**a**-**f**) Whole-mount *in situ* hybridization indicated diffused expression of *cyp2aa9* in the mesoderm and tail region (arrowheads) at 20 hpf and 36 hpf (**a**, n=14/17; **b**, n=15/17). Dashed boxs were enlarged in **c**-**d**, which is controlled by the sense probe as shown in **e**-**f**.

Supplementary Video Legends

Supplementary Video 1. Real-time imaging of budding HSCs (arrowheads) in the *Tg(cmyb:GFP, kdrl:mCherryRas)* transgenic embryos injected with *conMO* from 26 hpf to 30 hpf. The durations of the imaging were 4 hours. Scale bar, 50 μm. Related to Fig. 1i, k.

Supplementary Video 2. Real-time imaging of budding HSCs (arrowheads) in the *Tg(cmyb:GFP, kdrl:mCherryRas)* transgenic embryos injected with *cyp2aa9MO* from 26 hpf to 30 hpf. The durations of the imaging were 4 hours. Scale bar, 50 μm. Related to Fig. 1j, l.

Supplementary Materials and Methods

MO and mRNA injections. The *p53MO*[23] were synthesized by the Gene-Tools, LLC. 5 ng *p53MO* plus 5 ng *conMO* or *cyp2aa9MO* were injected. *cyp2aa9-GFP* mRNAs were synthesized from the Not I-linearized *pCS2(+)* plasmids using the mMessage mMachine kit (Ambion) as previously described[42]. Injections were performed as previously described[43]. 70 pg of *cyp2aa9-GFP* mRNA were injected.
